# Kinetic, isotherm, and thermodynamic studies for adsorptive removal of basic violet 14 from aqueous solution

**DOI:** 10.55730/1300-0527.3502

**Published:** 2022-10-04

**Authors:** Yasemin TAMER, Hale BERBER

**Affiliations:** 1 Department of Polymer Material Engineering, Faculty of Engineering, Yalova University, Yalova, Turkey; 2 Department of Metallurgical and Materials Engineering, Faculty of Chemical and Metallurgical Engineering, Yıldız Technical University, İstanbul, Turkey

**Keywords:** Adsorption, basic violet 14, semi-IPN hydrogel, free radical polymerization, kinetics, isotherms, thermodynamics

## Abstract

In this study, the performance of chitosan based semi-IPN nanocomposite hydrogels for the adsorptive removal of basic violet 14 (BV14) from aqueous solution has been explored. Batch adsorption studies were conducted to determine the effect of various parameters on BV14 adsorption, and optimum values were reported as pH of 8, the adsorbent dosage of 0.025 g, initial BV14 concentration of 5 mg L^−1^ and contact time of 90 min at a temperature of 25 °C. The semi-IPN hydrogel containing 0.5% by weight GO showed the improved adsorption capacity for BV14 compared to the neat hydrogel adsorbent, and the maximum adsorption capacity was 276.21 mg g^−1^ with a removal efficiency of 90.4%. Kinetic studies have shown that the pseudo-second-order kinetic model can well describe BV14 adsorption. The equilibrium adsorption data showed the best fit with the Langmuir model. Thermodynamic studies revealed that BV14 adsorption was physical in nature and occurred spontaneously and endothermically. The BV14 removal efficiency above 84% was obtained after five consecutive adsorption-desorption cycles, which has proved the reusability performance of the adsorbent and the recovery potential of BV14 dye. Overall, the results of this study indicated that GO containing chitosan based semi-IPN nanocomposite hydrogel could be an effective and environmentally friendly adsorbent for the successful removal of BV14 molecules from aqueous solution.

## 1. Introduction

Water, one of the indispensable essential elements of life, is a critical resource for the survival of all living organisms, healthy ecosystems, food production and the sustainability of the economy [1]. In recent years, some water resources have been consumed rapidly and unconsciously with uncontrolled industrialization, and some are polluted and become unusable and harmful to the environment. The reduction of vital water resources and quality directly affects public health and living standards, and it may cause diarrhea, typhoid fever, cholera, and the further spread of the Covid-19 global epidemic that we are experiencing on a global scale [1–3].

Water resources are directly or indirectly polluted by detergents, insecticides and herbicides, several organic and inorganic chemicals, heavy metals, dyes, chemical wastes, etc., resulting from various activities of human beings such as urbanization, population growth and industrial production [4]. These pollutants released uncontrollably to natural water resources, which are of great importance for the continuity of life, cause irreversible damage to the health of all terrestrial and sea creatures. Among them, dyes discharged from various industries such as textile, leather, printing, plastic, and food industries are one of the most crucial water pollutants [5]. Dyes can be in two main structures, inorganic in nature and organic compounds mainly consisting of conjugated phenyl structures used in the dyeing industry [6]. Approximately 15% of the synthetic organic dyes produced worldwide are lost during the dyeing processes and are released into the living ecosystem with industrial wastewater [7]. These dye molecules, which cause serious pollution problems in water bodies, are highly colored and can affect the photosynthetic activity in aquatic life by inhibiting sunlight penetration and consuming dissolved oxygen [8]. In addition, most dyes are highly toxic and carcinogenic, and the accumulation of these dye molecules in some aquatic organisms creates a serious environmental problem and threatens human health by causing skin irritation, allergic dermatitis, mutations and cancer.

Dyes are grouped into three broad classes according to their ionic charge as being anionic (direct, acidic and reactive dyes), cationic (basic dyes) and nonionic (disperse dyes) [9]. Among them, cationic dyes, also known as basic dyes, are highly visible even at very low concentrations due to their high brightness and color intensities and are known to be highly toxic compared to other dyes [10]. Basic violet 14 (BV14), also known as basic fuchsin, belongs to the tri-phenyl methane dye group, commonly known as intense colored dye molecules, and is widely employed in the textile and leather industries and is also used as a dermatological agent for staining collagen, muscle, or mitochondria [10]. It has been reported that BV14 is readily absorbed through human skin and has a cytotoxicity on human red blood cells. Thus, inhalation of BV14 dye may irritate the respiratory tract and damage organs such as blood, liver, thyroid, spleen and pituitary, and physical contact with this dye molecule may cause a gastrointestinal irritation [11].

Due to the toxic nature and hazardous effects of the basic dyes, it is crucial to treat the industrial wastewater containing these dye molecules at the source before being discharged into the living ecosystem. These dye molecules are also resistant to environmental conditions such as light, oxidizing agents or aerobic digestion because of their complex aromatic structure and synthetic origin, making them difficult to biodegrade. So far, various chemical and physical methods such as chemical precipitation, chemical oxidation, ion exchange, filtration, electrolysis, adsorption and biological treatment systems or a combination of these methods have been applied to remove such pollutants effectively and quickly from wastewater [12–14]. However, most of these methods have significant energy requirements, and their usage is limited due to their high economic and environmental costs. In addition, none of these methods successfully completely removes these toxic dyes from wastewater [15]. Adsorption is a frequently preferred method for removing dye molecules from wastewater due to its high efficiency, low cost, easy industrial application and environmentally friendliness [16]. Another important advantage of the adsorption method is the absence of sludge formation, which is one of the most significant factors increasing the operating costs of traditional wastewater treatment plants and is difficult to dispose of. In addition, removing pollutants in industrial wastewater at their source by the adsorption process will allow the reuse of wastewater and the recovery of chemical substances separated from the wastewater. Thus, by releasing fewer pollutants into the aquatic environment, the quality of existing water resources will be increased, and significant water savings will be achieved.

In adsorption applications, minerals, inorganic synthetic materials, synthetic polymers, or their combinations have been widely used as adsorbent materials [17–19]. It is expected that the adsorbent material will exhibit high adsorption efficiency in a relatively short time, can be easily separated from wastewater after the adsorption process without causing secondary pollution, and be environmentally friendly. Polymeric adsorbents have been frequently used in adsorption applications due to their pore size and distribution, large surface area, adjustable surface chemistry, superior mechanical properties, and regeneration properties [20,21]. Different polymeric adsorbents such as microspheres, hydrogels, resins, and sol-gels have been designed and developed to provide a high adsorption capacity [22]. In recent years, crosslinked porous polymeric hydrogels have received a great attention due to their high swelling ability, surface area sensitivity to pH, adsorption capacities, low cost, reusability, and the ability to be produced with the desired strength [23]. These polymeric hydrogels, which have numerous functional groups such as hydroxyl, carbonyl, carboxyl, sulfonyl, and amide, can efficiently adsorb dye molecules from wastewater through hydrogen bonding, van der Waals interactions, electrostatic interactions and hydrophobic interactions, as well as having high swelling capacities.

Polymeric hydrogels can be prepared from polysaccharide-based biopolymers such as chitosan (CS) and its derivatives. Chitosan, the second most abundant polysaccharide globally, can be obtained by deacetylation of chitin and can be used as an effective adsorbent due to its hydroxyl and amino functional groups. In addition, its nontoxicity, biodegradability, and biocompatibility, along with easy availability and low cost, make it indispensable for environmental applications [24,25]. Various biocompatible functional monomers can be used to obtain CS-based hydrogels with favorable chemical and physical properties. Acrylamide, one of the leading monomers used in hydrogel preparation, and itaconic acid with double carboxylic acid groups can be used to adjust the swelling properties and mechanical strength of CS-based hydrogels and to improve their adsorption capacity towards cationic dyes [26,27]. In addition, nanocomposite hydrogels can be prepared by uniformly dispersing graphene oxide (GO) in the hydrogel matrix to enhance various properties, such as poor mechanical strength [28].

The objective of this study is to design a polymeric hydrogel as an efficient, environmentally friendly, and high adsorption capacity adsorbent for the removal of basic violet 14 from aqueous solution. Semi-IPN structured and GO doped nanocomposite hydrogels were prepared by copolymerizing acrylamide and itaconic acid monomers in the presence of CS biopolymer, and to the best of our current knowledge, this design has not been used in BV14 dye adsorption applications. The effect of different parameters such as contact time, initial pH, adsorbent dosage, initial dye concentration and temperature on BV14 adsorption was investigated. To evaluate the adsorption mechanism of BV14 on nanocomposite hydrogel, different kinetic and isotherm models were applied to the experimental adsorption data, and also thermodynamic studies were carried out. In addition, the reusability of the nanocomposite was also studied by sequential adsorption-desorption cycles.

## 2. Materials and methods

### 2.1. Materials

**Figure 1:**
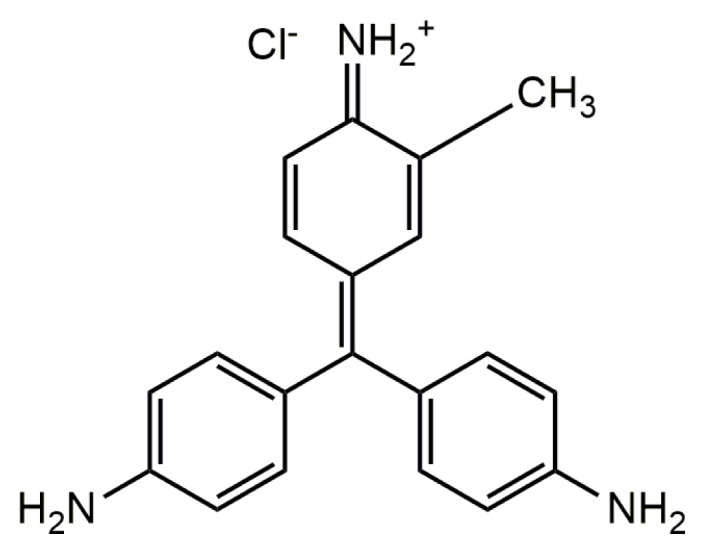
Chemical structure of basic violet 14 (BV14) (C_20_H_19_N_3_HCl, M_W_: 337.85 g mol^−1^, λ_max_: 545 nm).

The monomers, itaconic acid (IA, 99%) and acrylamide (AM, 99%), catalyst, N,N,N′,N′-tetramethylethylenediamine (TEMED, 99%) and acetic acid (AA, 100%) were supplied from Merck. Chitosan (CS, medium viscosity) from crab shells with a degree of deacetylation of 80%, initiator, ammonium persulfate (APS, 98%) and basic dye, basic violet 14 (BV14, ≥80) were obtained from Sigma-Aldrich. N,N′-methylenebis (acrylamide) (MBA, 96%) was obtained from Acros Organics. Graphene oxide was obtained as explained in our previously reported procedure [29]. A stock solution of BV14 was prepared by dissolving BV14 in distilled water, and dye solutions of various concentrations were prepared by diluting this stock solution. HCl (0.1 M) and NaOH (0.1 M) were also obtained from Merck and used to adjust the medium pH. The chemical structure of BV14 dye is given in Figure 1.

### 2.2. Methods

#### 2.2.1. Preparation of hydrogels

To synthesize the hydrogel adsorbent, chitosan (0.1 g) was first dissolved in 5 mL of 1 wt% acetic acid solution and stirred overnight. APS (0.022 g) was dissolved in 1 mL distilled water and added to this solution. On the other hand, predetermined amounts of GO were dispersed in double distilled water by ultrasonication and then AM (0.9 g), IA (0.1 g) and MBA as crosslinker (0.03 g) were added to this GO dispersion and stirred until monomers were completely dissolved. These two solutions, which were prepared separately, were brought together, and the resulting mixture was stirred for 2 h under argon atmosphere. Afterwards, TEMED (0.0112 g) was dissolved in this mixture, and the obtained homogeneous mixture was transferred into a polymeric tube and kept at 60 °C overnight in a water bath to form a hydrogel structure. Afterwards, TEMED (0.0112 g) was dissolved in this mixture, and the obtained homogeneous mixture was transferred to a cylindrical tube and placed in a water bath at 60 °C overnight to induce free radical initiated polymerization of AM and IA in the presence of chitosan. To remove unreacted components, the hydrogel was extracted with distilled water for 6 h and then freeze-dried for 72 h. The obtained semi-IPN hydrogel was designated as CS/(AM-co-IA)/GO. Also, the neat semi-IPN hydrogel, CS/(AM-co-IA) without GO was prepared with the above procedure.

#### 2.2.2. Characterization

The structure of the freeze-dried CS/(AM-co-IA) and CS/(AM-co-IA)/GO nanocomposite hydrogels was determined by Fourier-transform infrared (FT-IR) spectra in the wavelength range of 400–4000 cm^−1^ with a Thermo Scientific Nicolet iS10 FTIR spectrophotometer equipped with an ATR (Attenuated Total Reflection) apparatus. The morphology of the freeze-dried hydrogels was investigated using a Zeiss Evo LS scanning electron microscopy (SEM) operated with an accelerating voltage of 10 kV. The crosssectional micrographs of the samples were obtained after coating with palladium gold alloy. X-ray diffraction (XRD) patterns were recorded with PANalytical Empyrean X-ray diffractometer using 45 kV, 30 mA, Cu Kα radiation (λ =1.54056 Å) with a scan speed of 2*θ* = 1° min^−1^ in the scan range from 4° to 80° 2*θ*.

#### 2.2.3. Dye adsorption studies

The adsorption behavior of CS/(AM-co-IA) and CS/(AM-co-IA)/GO hydrogels towards BV14 dye was investigated under various experimental conditions, such as initial pH (2 to 12), adsorbent dosage (0.025 to 0.1 g), initial BV14 dye concentration (1.0, 3.0, 5.0, 10.0, 15.0, and 20.0 mg L^−1^), contact time (from 0 min to 210 min) and temperature (25, 35, and 45 °C). The stock solution of BV14 dye was prepared at a concentration of 5 mg L^−1^ by using double distilled water. Batch adsorption studies were carried out in 50 mL of BV14 dye solution by shaking for 3 h at 120 rpm in a shaker at predetermined adsorption conditions (Scheme). Then, the hydrogel adsorbent was taken out from the BV14 dye solution and the absorbance of the remaining BV14 dye solution was measured using a Shimadzu, UV 1800, double beam UV-Vis spectrophotometer at 541 nm, maximum absorption wavelength of BV14. The pH value of BV14 solution in the range of 2–12 was adjusted using 0.1 M NaOH and 0.1 M HCl. All adsorption experiments were performed twice. A standard linear absorbance calibration curve was plotted against various dye solution concentrations to calculate the amount of dye in the solutions. The adsorption capacity of the hydrogel, the amount of BV14 adsorbed at equilibrium, was calculated by using Eq. 1.


(1) $$q_{e}=\frac{(C_{0}-C_{e}).V}{w},$$

where *q*_e_ (mg g^−1^) is the adsorbed amount of BV14 per gram adsorbent, C_0_ (mg L^−1^) and C_e_ (mg L^−1^) are BV14 dye solution concentration at the initial and equilibrium stages, respectively. *V (L)* is the volume of the BV14 solution, and w (g) is the amount of the hydrogel adsorbent.

The removal efficiency (%) of BV14 was calculated by using Eq. 2.


(2) $$\%\;RE=\frac{\left(C_{0}-C_{e}\right)}{C_{0}}\times 100$$

#### 2.2.4. Reusability studies

After the adsorption process, the BV14 loaded hydrogel sample was removed from the adsorption medium and treated with 0.01 M HCl to induce desorption of BV14. Then, the hydrogel sample was immersed in 50 mL of 0.01 M NaOH for regeneration and washed with distilled water until neutralized. After freeze-drying, the resulting regenerated hydrogel was applied to the next adsorption cycle. Five consecutive adsorption-desorption regeneration cycles were applied to test the reusability performance of the hydrogel adsorbent.

## 3. Results and discussion

### 3.1. Characterizations

**Figure 2:**
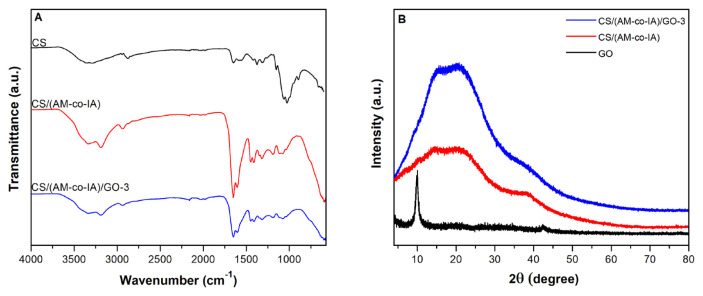
(A) FTIR-ATR spectra of pure chitosan, neat CS/(AM-co-IA) and CS/(AM-co-IA)/GO-3 hydrogel and (B) XRD spectra of GO, neat CS/(AM-co-IA) and CS/(AM-co-IA)/GO-3 hydrogel.

The neat CS/(AM-co-IA) hydrogels with the semi-IPN structure were synthesized by free radical copolymerization of the acrylamide and itaconic acid in the presence of chitosan. CS/(AM-co-IA)/GO nanocomposite hydrogels were also prepared by the same procedure with the addition of 0.1%, 0.3%, and 0.5% by weight GO nanosheets to this network structure. To confirm the chemical structure of the CS/(AM-co-IA)/GO semi-IPN hydrogels, FTIR analysis was conducted, and the infrared spectra of CS, neat CS/(AM-co-IA) hydrogel and CS/(AM-co-IA)/GO-3 nanocomposite hydrogel are given in Figure 2A. The major IR bands in the spectra of CS at 3360, 3295, 2944, 2879, 1652, 1590, 1567, 1318, 1420, 1375, 1150, 1063, and 1028 cm^−1^ were assigned to O-H stretching, N-H stretching, C-H asymmetric and symmetric stretching, C=O stretching of amide I, N-H bending of −NH_2_ groups, N-H bending of amide II and C-N stretching of amide III, C-H bending, O-H bending, C-O-C asymmetric stretching and C-O stretching vibrations of the saccharide structure of CS, respectively [30,31]. The infrared spectra of CS/(AM-co-IA) indicate distinctive absorption bands at 3342 and 3194 cm^−1^ due to the stretching vibrations of O-H and N-H groups. The absorption band at 1605 cm^−1^ can be attributed to N-H bending, which is characteristic of acrylamide and chitosan. The observed band at 1651 cm^−1^ can be attributed to the C=O stretching vibration of the carboxylic groups of itaconic acid. In addition, C-H bending and C-N stretching vibrations appear at 1416 and 1320 cm^−1^. Moreover, the C-O stretching vibration of COO^−^ groups of itaconic acid appears at 1448 cm^−1^. As seen from the FTIR spectrum of CS/(AM-co-IA)/GO-3 nanocomposite hydrogel, there is no notable change in the characteristic absorption band positions with the incorporation of GO into the network structure; however, observing a decrease in the intensities of the characteristic bands and the broadening of some peaks can be ascribed to the formation of hydrogen bond between the oxygen-containing groups of GO nanosheets and polymer chains [29,32].

The crystalline structure of the GO and semi-IPN hydrogel was evaluated by XRD analysis. As shown in Figure 2B, the crystal structure of GO was confirmed by the characteristic narrow and sharp peak at 2θ = 10.08° and a rather weak peak at 2θ = 42.34°. In the XRD pattern of the neat CS/(AM-co-IA) semi-IPN hydrogel, the two broad peaks observed at 2θ = 20.98° and 38° can be attributed to the amorphous nature of the hydrogel. On the other hand, the CS/(AM-co-IA)/GO-3 nanocomposite hydrogel showed the same XRD pattern with a broad and more intense diffraction peak at 2θ = 20.52° and 2θ = 37.22°, indicating the amorphous structure of the nanocomposite and the good dispersion of GO nanosheets in the semi-IPN hydrogel.

**Figure 3:**
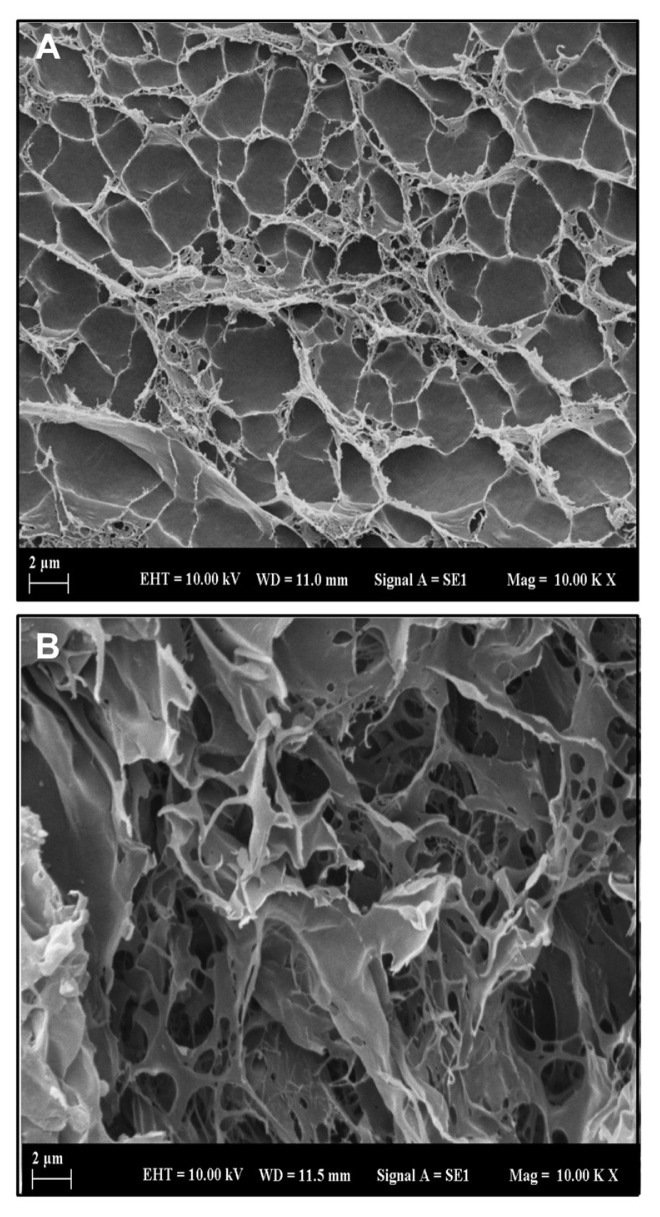
SEM micrographs of (A) CS/(AM-co-IA) and (B) CS/(AM-co-IA)/GO-3 hydrogels.

The morphologies of the semi-IPN hydrogel samples were observed with SEM, and crosssectional images of the neat and GO containing CS/(AM-co-IA) hydrogels are presented in Figure 3. As shown in Figure 3A, the neat CS/(AM-co-IA) hydrogel has a thin-walled, interconnected microporous structure over a wide range of sizes and small pores around 100 nm. On the other hand, CS/(AM-co-IA)/GO-3 hydrogel containing 0.5% by weight of GO exhibited a significantly different pore morphology due to the contribution of GO nanosheets that act as a physical crosslinker by forming hydrogen bond interactions between polymer chains and functional groups of GO (Figure 3B). As a result of these interactions, an irregular porous structure with a small pore size as well as large holes and also sheet-like formations with smooth surfaces was observed in the SEM image of the CS/(AM-co-IA)/GO nanocomposite hydrogels that were also prepared by the same procedure with the addition of 0.1%, 0.3%, and 0.5% by weight GO nanosheets to this network structure [33].

### 3.2. The parameters affecting the adsorption process

**Figure 4:**
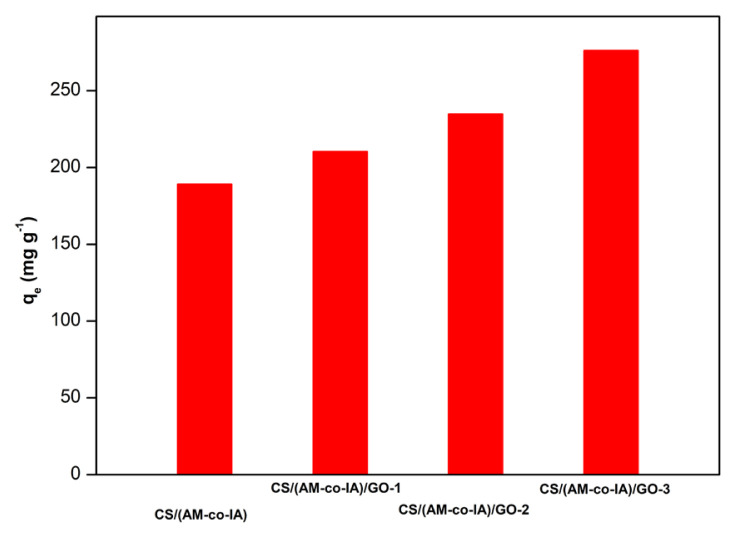
Adsorption capacities of CS/(AM-co-IA) hydrogel and its GO containing nanocomposites for removal of BV14.

The adsorption capacities of the neat CS/(AM-co-IA) hydrogel and its GO containing nanocomposites were examined for the removal of BV14 at an initial dye concentration of 5 mg L^−1^. The removal efficiencies of the CS/(AM-co-IA) hydrogels are presented in Figure 4 comparatively, and as seen, both hydrogels exhibited excellent adsorptive removal capacities. The adsorption capacity of neat CS/(AM-co-IA) hydrogel was 189.23 mg g^−1^ with an adsorption efficiency of 75.2%, whereas CS/(AM-co-IA)/GO-1, CS/(AM-co-IA)/GO-2 and CS/(AM-co-IA)/GO-3 nanocomposite hydrogels achieved 81.2%, 86.3%, and 90.4% adsorption efficiencies with adsorption capacities of 210.45, 234.85 and 276.21 mg g^−1^, respectively. This result demonstrates that the addition of GO and increasing its ratio in the network structure improved the dye adsorption capacity of the semi-IPN hydrogel due to its several surface-active oxygen containing functional groups. Therefore, CS/(AM-co-IA)/GO-3 nanocomposite hydrogel containing 0.5 wt% GO and exhibiting the highest adsorption capacity was chosen for further experimental studies.

#### pH effect

**Figure 5:**
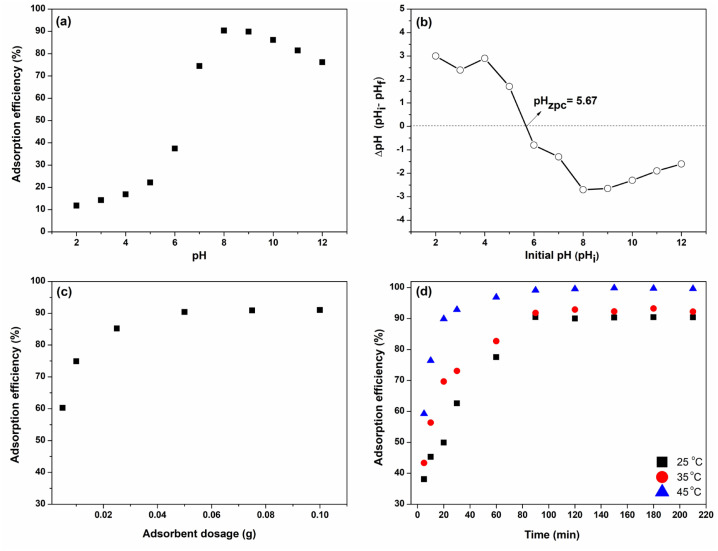
(a) Zero point charge (pH_ZPC_) of CS/(AM-co-IA)/GO-3 nanocomposite hydrogel, and effects of (b) initial pH, (c) adsorbent dosage and (d) contact time-temperature on the adsorption of BV14 by CS/(AM-co-IA)/GO-3.

CS/(AM-co-IA)/GO-3 hydrogel possesses various surface functional groups such as hydroxyl, carboxyl and amine, which are responsible for the interactions between dye molecules and the adsorbent because of the protonation and deprotonation of these groups depending on the pH of the adsorption medium. As shown in Figure 5b, the dye adsorption efficiency was found to be very low, as 11.8% at pH 2. Under this strongly acidic pH condition, excessive amount of H^+^ ions competing with the BV14 dye molecules may have resulted in lower dye uptake. A slight increase in adsorption efficiency was detected in the pH range from 2 to 6, followed by a remarkable increase with increasing pH values. The maximum uptake was reported as 276.21 mg mL^−1^ at pH 8 with a removal efficiency of 90.4%. This behavior can be explained by the zero-charge point (pH_ZPC_), at which the net surface charge of the adsorbent surface was zero, and this value was 5.67 for the CS/(AM-co-IA)/GO-3 hydrogel (Figure 5a). At pH < pHzpc, the adsorbent surface became positively charged due to the ionization of amine groups to form NH_3_^+^ groups, resulting in lower BV14 removal efficiencies. At pH > pHzpc, the ionization of carboxyl groups makes the adsorbent surface have a net negative charge, and the formation of strong electrostatic interactions between cationic BV14 dye molecules and the adsorbent surface improves the adsorption efficiency. The observed decrease in BV14 adsorption after pH 10 can be explained by the screening effect of excess Na^+^ ions of NaOH competing with dye molecules.

#### Adsorbent dosage effect

The adsorption of BV14 on the CS/(AM-co-IA)/GO-3 hydrogel was studied with varying dosages of adsorbent from 0.005 g to 0.100 g at a fixed initial concentration of 5 mg L^−1^ at a constant volume of 50 mL, at pH 8 and 25 °C. It is clear from Figure 5c that the initial dye removal percentage was low but increased as the adsorbent dosage increased and became almost constant after a certain time. The rising trend in BV14 dye removal efficiency can be ascribed to the increased surface area as well as the vacant dye-binding sites present on the adsorbent surface with the increase in adsorbent dosage. Increasing the adsorbent dosage from 0.025 g to 0.100 g results in a very little rise in removal efficiency due to the saturation of free active sites that BV14 dye adsorption can take after the optimum amount of adsorbent. When all the results were considered, the optimum adsorbent dosage was determined as 0.025 g for further adsorption studies.

#### BV14 dye concentration effect

To investigate the initial BV14 concentration effect, the adsorption studies were performed using 0.025 g CS/(AM-co-IA)/GO-3 hydrogel and BV14 dye solutions in the concentration range of 1.0 mg L^−1^ to 20 mg L^−1^. A gradual decrease in adsorption percentage from 90.4% to 79.6% was detected by increasing the initial BV14 dye concentration. This trend can be explained by the fact that at low dye concentrations, rapid adsorption occurs since the free dye-binding parts on the adsorbent surface are significantly higher than the number of BV14 dye molecules, whereas at higher BV14 concentrations, the decrease observed in the removal percentage may be due to the complete coverage of the free adsorption sites on the adsorbent surface. On the other hand, the adsorption capacity increased from 137.90 mg g^−1^ to 276.21 mg g^−1^ by increasing the initial BV14 concentration from 1.0 mg L^−1^ to 20 mg L^−1^. The formation of strong interactions between BV14 molecules and the vacant active sites of the adsorbent, increasing the mass transfer driving force, may give an explanation for this increase.

#### Temperature effect

The rate of BV14 adsorption was investigated with a temperature range of 25, 35, and 45 °C. As seen in Figure 5d, the adsorption capacity at equilibrium was increased significantly from 276.21 mg g^−1^ at 25 °C to 334.48 mg g^−1^ at 45 °C. The favored uptake of BV14 dye with increasing temperature can be attributed to the fact that increasing the temperature leads to an increase in the molecular mobility of the dye molecules, and thus, the diffusion rate, as well as enhanced interactions between the empty spaces on the adsorbent surface and the BV14 dye molecules.

#### Contact time effect

The influence of contact time on the adsorption of BV14 dye was investigated using 0.025 g adsorbent at a constant BV14 dye concentration of 5 mg L^−1^, a constant initial pH of 8, and a contact time range of 0–210 min at different temperatures and the established trends were given in Figure 5d. At the beginning of the adsorption process, the removal efficiencies were fast due to the higher amount of vacant binding sites on the adsorbent surface, favoring more BV14 adsorption. As the contact time increased, the vacant adsorption sites began to fill, and dye removal reached an almost constant value within 90 min. Moreover, the existence of possible repulsive forces between BV14 molecules remaining in bulk solution and that was adsorbed by the hydrogel made it difficult to occupy the remaining unfilled sites [34]. As a result, the adsorption of BV14 dye onto CS/(AM-co-IA)/GO-3 adsorbent can be evaluated in two distinct stages, which are relatively fast, followed by slow, and the optimum contact time for the BV14 adsorption process was determined as 90 min. The observed trend is in good agreement with the literature [4].

### 3.3. Kinetics of BV14 adsorption

The adsorption process generally includes several stages: first, penetration of adsorbate molecules from the aqueous solution to the surface of the adsorbent; second, diffusion of adsorbate molecules from the outer surface to inner adsorption sites; and third, the interaction between the reactive sites of adsorbent and the adsorbate molecules through hydrogen bonds, electrostatic interactions, ion-exchange, hydrophobic attractions, chemical bonding, and so on [35]. Therefore, evaluating adsorption kinetics is of primary importance in determining the adsorption mechanism. To investigate the kinetics of BV14 removal, pseudo-first-order [36] and pseudo-second-order [37] kinetic models were tested by fitting the experimental data as shown in Eqs.3 and 4, respectively.


(3) $$\text{ln}(q_{e}-q_{t})=lnq_{e}-k_{1}t$$

(4) $$\frac{\text{t}}{{\text{q}}_{\text{t}}}=\frac{1}{{\text{k}}_{2}\;{{\text{q}}_{\text{e}}}^{2}}+\frac{1}{{\text{q}}_{\text{e}}}\text{t}$$

In these equations, q_t_ and q_e_ (mg g^−1^) are the amounts of BV14 adsorbed at various times t and equilibrium, k_1_ (min^−1^) and k_2_ (g mg^−1^ min^−1^) are the pseudo-first-order and pseudo-second-order rate constants, respectively.

**Figure 6:**
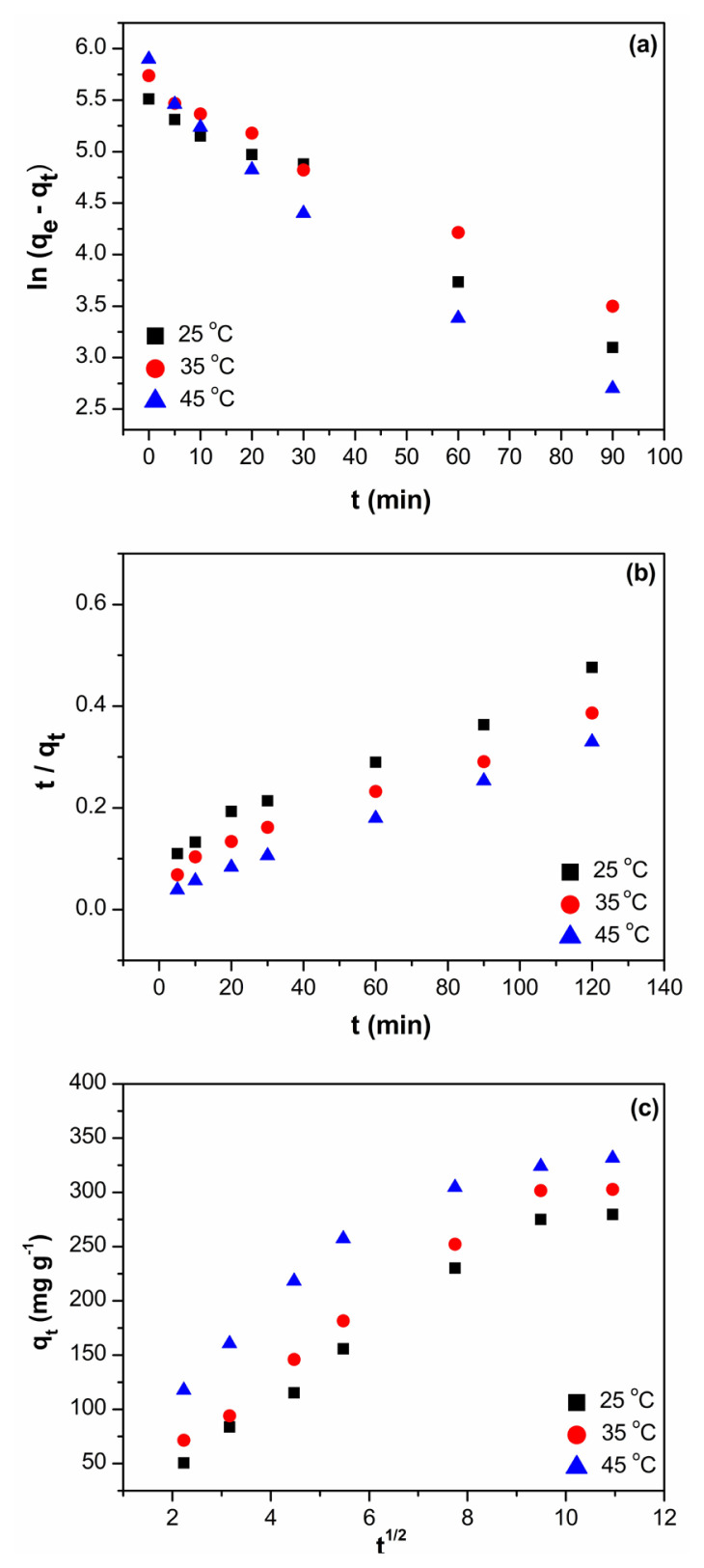
(a) Pseudo-first-order, (b) pseudo-second-order, and (c) intraparticle diffusion kinetic plots for the adsorption of BV14 onto CS/(AM-co-IA)/GO-3 (pH:8, C_o_:5 mg L^−1^ V:50 mL, m: 0.025 g).

By using the experimental data, the linear curves of ln (q_e_ – q_t_) against time t, and t/q_t_ against t were drawn for pseudo-first-order and pseudo-second-order models, respectively, as given in Figures 6a and 6b. Accordingly, the kinetic parameters, q_e_, k_1_, and k_2_ for the adsorption of BV14 were calculated from the plots and displayed in Table 1. Comparing the correlation coefficients of these kinetic models, it was seen that the kinetics of BV14 adsorption onto the nanocomposite hydrogel followed pseudo-second-order model. This means that BV14 adsorption process is mainly controlled by chemisorption via electron sharing or electron exchange with the formation of covalent forces between the hydrogel adsorbent and dye molecules. In addition, the endothermic nature of the adsorption process was proved by the increased rate constants of the pseudo-second-order kinetic model with temperature, which can be assigned to the formation of more active sites for BV14 adsorption due to the breaking of bonds on the adsorbent surface with increasing temperature [38]. The trend obtained appears to be in good agreement with the literature as reported for basic violet 14 adsorption onto TOC-KMnO_4_ adsorbent [39].

In addition, the intraparticle diffusion model [40], which assumes that the internal diffusion of dye molecules is the rate-limiting step, was also applied to the experimental data to further analyze the adsorption kinetic of BV14 as expressed by Eq. 5,


(5) $$q_{t}=k_{3}\;t^{1/2}+C,$$

where k_3_ (mg g^−1^ min^−1^) is the rate constant of intraparticle diffusion model and C represents the intercept related to the thickness of the boundary layer.

Figure 6c shows the linear plot of q_t_ against t^1/2^ plotted by fitting the experimental BV14 adsorption data to the intraparticle diffusion model. The fact that the regression of this plot is linear and passes through the origin indicates that the adsorption rate is controlled by intraparticle diffusion throughout the adsorption process [41]. The plot given in Figure 6c showed an initially curved and then a linear section, attributable to bulk diffusion and intraparticle diffusion, respectively, and then equilibrium was reached, as can be confirmed by the observed plateau. Also, none of the graphs drawn at different temperatures passed from the origin, suggesting the existence of boundary layer control to some extent in the adsorption process. These results confirmed that the intraparticle diffusion model was not the only rate-limiting step, and the adsorption of BV14 may be affected by other processes.

### 3.4. Adsorption isotherm studies

**Figure 7:**
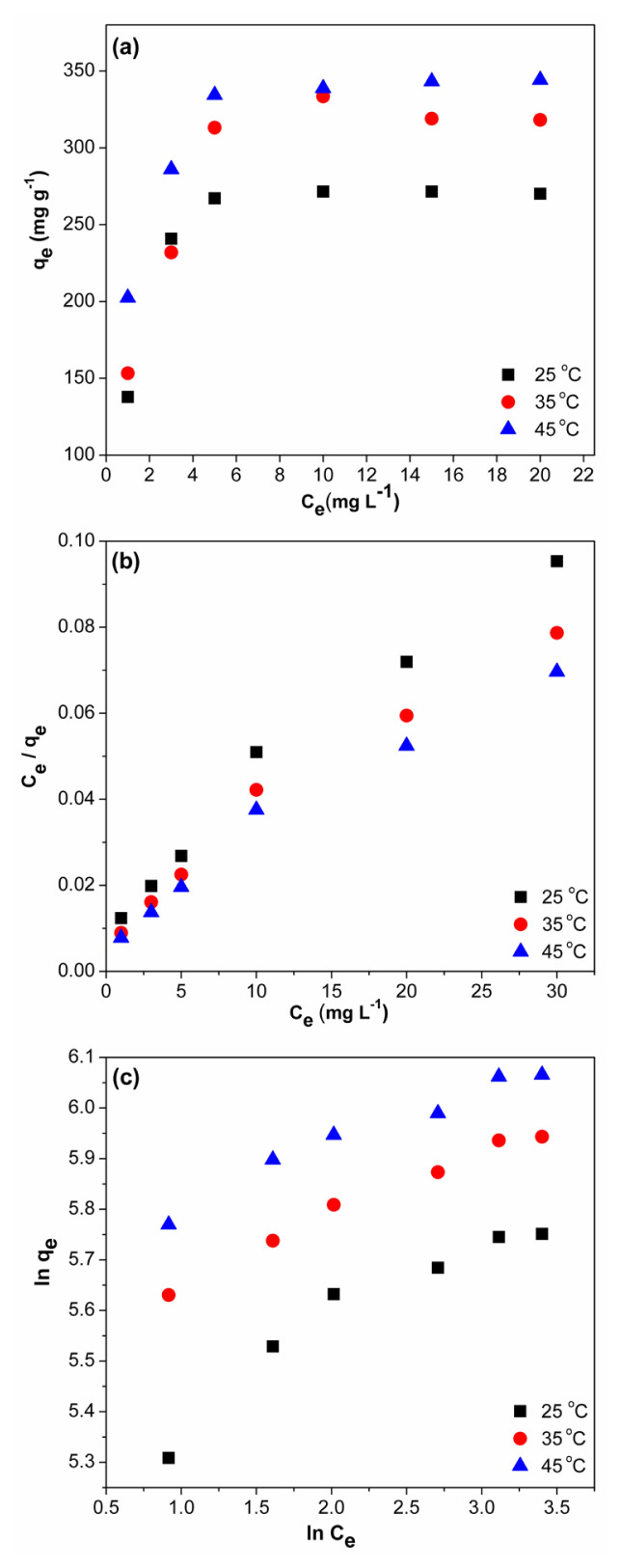
(a) isotherm curves, (b) Langmuir, and (c) Freundlich adsorption isotherm plots for the adsorption of BV14 onto CS/(AM-co-IA)/GO-3 (pH:8, V:50 mL, m: 0.025 g).

Adsorption isotherms are widely used to characterize the interactions between adsorbate and adsorbent and explain the distribution of adsorbate molecules between solid and liquid phases when the adsorption equilibrium is reached. Figure 7a indicates the isotherm curves of BV14 adsorption on CS/(AM-co-IA)/GO-3 at the temperatures studied. As can be seen, the adsorption capacity increases with temperature due to the breaking of the bonds on the adsorbent surface with increasing temperature and the formation of new free adsorption sites. This increase in adsorption capacity clearly indicates that the adsorption of BV14 onto hydrogel adsorbent is endothermic. Moreover, the obtained adsorption isotherm curves of BV14 can be classified as L type adsorption characterized by an initially relatively low adsorption capacity followed by increasing adsorption, which then remains constant [42].

To identify the nature of the BV14 removal process more clearly, the adsorption process was also studied by using (Eq. 6) [43] and Freundlich (Eq. 7) [44] isotherm models.


(6) $$\frac{C_{e}}{q_{e}}=\frac{1}{q_{m}K_{L}}+\frac{C_{e}}{q_{m}}$$

(7) $$\text{ln }q_{e}=\text{ln }K_{F}+\frac{1}{n}\;\text{ln }C_{e},$$

where q_m_ (mg g^−1^) and q_e_ (mg g^−1^) are the maximum adsorption capacity and equilibrium adsorption capacity, respectively, C_e_ (mol L^−1^) is the BV14 concentration at equilibrium, and K_L_ (L mg^−1^) and K_F_ (L mg^−1^) are the Langmuir and Freundlich constants, respectively. The dimensionless “n” factor describes the intensity of the Freundlich adsorption process, and its value changes by surface heterogeneity. “n” values greater than or equal to 1 indicate the linear and chemical nature of adsorption, while n values less than 1 indicate physical adsorption [45].

Also, the Langmuir isotherm can be characterized in more detail with the separation factor R_L_ (Eq. 8), which is generally expressed as follows:


(8) $$R_{L}=\frac{1}{1+K_{L}\;C_{0}},$$

where C_0_ (mol L^−1^) is the initial BV14 concentration. R_L_ values equal to zero, equal to unity or greater than 1 indicate the irreversible, linear or unfavorable nature of the adsorption, respectively, while favorable adsorption can be defined with R_L_ values between zero and one.

Using the experimental data, the linear plots of C_e_/q_e_ versus C_e_ were plotted (Figure 7b) and the kinetic parameters of the Langmuir model, q_m_ and K_L_, were obtained from the slope and the intersection point of this linear plot, respectively and are given in Table 2. For Freundlich model, the linear ln q_e_ versus ln C_e_ plot was drawn (Figure 7c), and the kinetic parameters, n and K_F_, were obtained from the slope and the intersection point of this linear plot, respectively, and are given in Table 2.

Considering the regression coefficients of these two isotherm models (Table 2), it can be said that the experimental adsorption data fit well with the Langmuir model, which has high correlation values (r^2^ > 0.99). This indicates the existence of monolayer adsorption on homogeneous surfaces where the adsorption of each dye molecule has equal activation energy, that is, once a dye molecule fills an empty adsorption site, no further adsorption occurs in this same site. Langmuir adsorption isotherm can be applied for all temperature values as can be seen from the obtained kinetic parameters, and the observed increase in q_m_ values with temperature can be attributed to the increase in free adsorption sites due to the breaking of bonds on the adsorbent surface. This also indicates that the adsorption proceeds endothermically. From the R_L_ values which were between zero and one, the favorable adsorption of basic violet 14 on CS/(AM-co-IA)/GO-3 hydrogel was confirmed. The lower correlation coefficients obtained for the Freundlich adsorption isotherm indicate that the BV14 adsorption process does not occur on heterogeneous surfaces such as multilayer adsorption as the Freundlich isotherm assumes. The obtained “n” values of the Freundlich isotherm model are between 1 and 10 for all temperatures showing the favorability of the BV14 adsorption. In addition, the adsorption of basic violet 14 on CS/(AM-co-IA)/GO-3 hydrogel followed a normal Langmuir isotherm, as can be seen from the 1/n values obtained for the temperatures studied [39].

### 3.5. Thermodynamic studies

**Figure 8:**
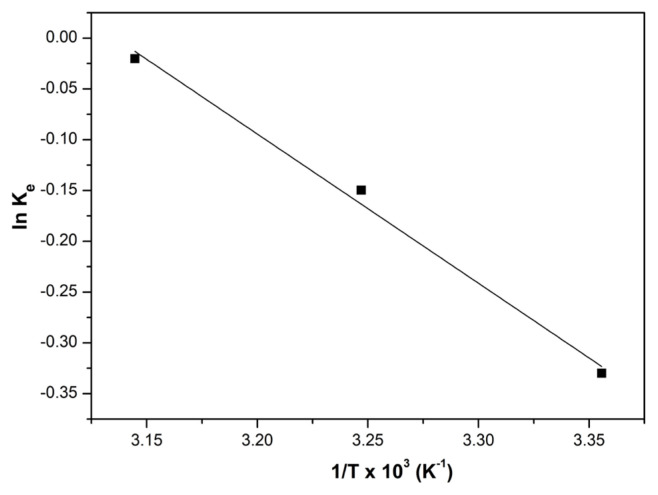
The plot of thermodynamic for the adsorption of BV14 onto CS/(AM-co-IA)/GO-3 (pH:8, C_o_:5 mg L^−1^ V:50 mL, m: 0.025 g).

The thermodynamic parameters provide important information about the energy changes related with the dye removal process and the nature of the adsorption process. Different energy conversions and transfers occur with the formation of physical or chemical interactions during the adsorption process, which can be explained by using thermodynamic parameters, notably, standard Gibbs free energy change (ΔG°), the enthalpy change (ΔH°) and entropy change (ΔS°) of the system [46]. Eqs. 9 and 10 given below can be used to calculate these parameters.


(9) $$\Delta G^{0}=-RT\;lnK_{e}$$

(10) $$\text{ln}\;K_{e}=\frac{\Delta S^{0}}{R}-\frac{\Delta H^{0}}{RT},$$

where K_e_ (L mg^−1^), T (K) and R (8.314 J mol^−1^ K^−1^) are the thermodynamic equilibrium constants which were determined from the Langmuir adsorption model, temperature and the universal gas constant, respectively. From the slope and intersection point of the ln K_e_ versus 1/T linear plot, the values of ΔH_0_ and ΔS_0_ can be obtained, respectively, that was given in Figure 8.

When the obtained values were examined (Table 3), the reported positive value of ΔH_0_ as 12.21 kJ kmol^−1^ confirms the endothermic nature of the adsorption process [47]. Also, the chemical or physical nature of adsorption can be expressed using enthalpy value, and since the obtained ΔH_0_ value is considerably smaller than 40 kJ kmol^−1^, it can be said that the dominating mechanism for BV14 adsorption on CS/(AM-co-IA)/GO-3 hydrogel is physisorption [48]. For the BV14 adsorption process, the entropy value was found to be positive as 38.31 J kmol^−1^ K^−1^, indicating the enhanced randomness of the interactions between dye molecules and the adsorbent surface during BV14 adsorption onto the hydrogel adsorbent [4]. Besides, the reported ΔG^0^ values were negative at all studied temperatures, affirming the spontaneity of BV14 adsorption. As depicted in Table 4, the detected increase in ΔG^0^ values with increasing temperature indicates that the interactions between BV14 molecules and active adsorption sites of the adsorbent surface increased at high temperatures [4].

Evaluation of the adsorption studies results shows that the adsorption of BV14 on CS/(AM-co-IA)/GO-3 occurs simultaneously with both physical and chemical adsorption. The kinetics and isotherm studies reveal the chemical adsorption process, while the thermodynamic analysis suggests physical adsorption. The porous structure and functional groups of the hydrogel contribute to physical adsorption through hydrogen bonding, weak Van der Waals forces, π-π interaction and electrostatic interactions between the cationic BV14 dye molecules and the negatively charged surface of the adsorbent. On the other hand, due to the endothermic nature of the adsorption process, the adsorption takes place better at higher temperatures, and as it is known, physical interactions such as van der Waals interactions and hydrogen bonding weaken with increasing temperature. Therefore, the adsorption capacity is expected to decrease with increasing temperature without chemical interactions. Therefore, it is clear that the adsorption process is also accompanied by chemical interactions between the active sites of the hydrogel adsorbent and BV14 dye molecules [49].

### 3.6. Reusability studies

**Figure 9:**
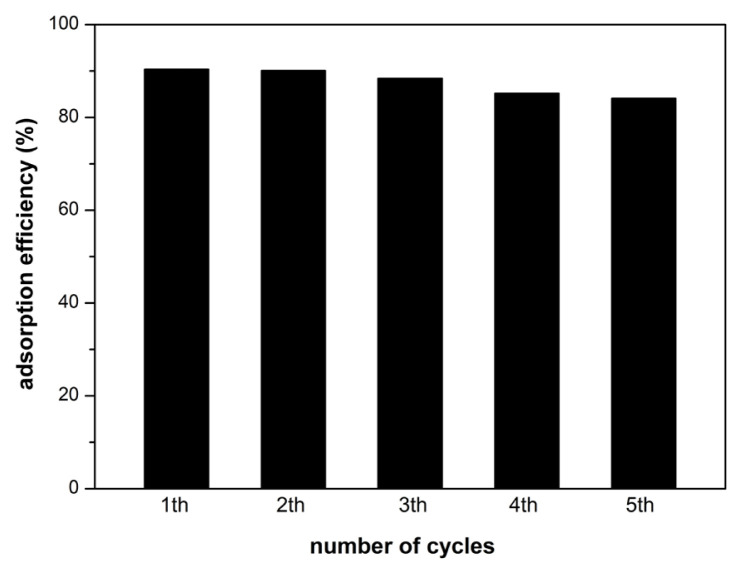
Adsorption capacity values of CS/(AM-co-IA)/GO-3 hydrogel in reusability study.

To confirm the adsorption performance of the adsorbent, five cycles of adsorption-desorption studies were conducted. Figure 9 shows the reusability trend of the CS/(AM-co-IA)/GO-3 nanocomposite hydrogel. The adsorption efficiencies were found to be 90.4%, 90.1%, 88.4%, 85.2%, and 84.1% in the first, second, third, fourth, and fifth cycles, respectively. This slight decrease observed in adsorption efficiency may be due to incomplete desorption of BV14 bound to the active sites of the adsorbent. However, the obtained high adsorption capacities suggest that even when used for up to five cycles, the nanocomposite hydrogel retains its structural integrity during the reclamation of BV14 and can be used in long-term adsorption applications.

### 3.7. Comparative studies

In order to determine the efficiency of CS/(AM-co-IA)/GO-3 nanocomposite hydrogel in removing BV14 dye, the maximum adsorption capacities of various adsorbents are given in Table 4 in comparison with the hydrogel adsorbent synthesized in this study. As shown in Table 4, the adsorption capacity value obtained for BV14 was much higher compared to other adsorbents already reported in the literature. Based on the higher adsorption capacity value obtained, it was seen that CS/(AM-co-IA)/GO-3 nanocomposite hydrogel can be used as a promising adsorbent for the removal of cationic BV14 dye from aqueous solution.

## 4. Conclusion

The present study demonstrated the design and efficacy of novel environmentally friendly chitosan based semi-IPN nanocomposite hydrogel adsorbents for removing basic violet 14 from aqueous solution. The results of batch adsorption studies clearly indicated that CS/(AM-co-IA)/GO-3 exhibited excellent adsorption ability toward BV14 and can remove 90.4% of the BV14 with a maximum adsorption capacity of 276.21 mg g^−1^ at pH 8 in 90 min with an adsorbent dosage of 0.025 g and an initial dye concentration of 5 mg L^−1^. The kinetic studies proved that the adsorption of BV14 fits the pseudo-second-order kinetic model with good correlation. Also, the monolayer adsorption of BV14 was proved by the applicability of the Langmuir model. Thermodynamic studies revealed the endothermic and spontaneous nature of the adsorption. The adsorption study results suggest that the adsorption of BV14 by CS/(AM-co-IA)/GO-3 is a combined physicochemical process. Reusability studies demonstrated that the CS/(AM-co-IA)/GO-3 hydrogel could successfully retain BV14 even after five adsorption-desorption cycles with a removal efficiency of 84.1%. Considering all the results, it can be stated that GO doped CS/(AM-co-IA) semi-IPN nanocomposite hydrogels with high adsorption capacity and reusability could be potentially used as a low-cost and effective adsorbent for successful removal of BV14 from aqueous solution.

## Figures and Tables

**Scheme:**
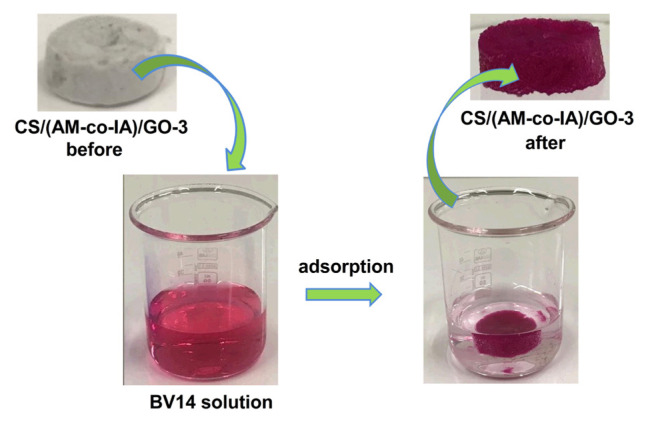
Schematic illustration of the BV14 adsorption process.

**Table 1:** Kinetic parameters for the adsorption of BV14 onto CS/(AM-co-IA)/GO-3.

Model	Parameters	Temperature		
		25 °C	35 °C	45 °C
Pseudo-first-order	**q** **_e_** ** (mg g** **^−1^** **)**	247.122	281.964	299.912
	**k** **_1_** ** (h** **^−1^** **)**	0.024	0.027	0.035
	**r** **^2^**	0.983	0.992	0.972
Pseudo-second-order	**q** **_e_** ** (mg g** **^−1^** **)**	289.017	354.610	390.625
	**k** **_2_** ** × 10** **^−4^** ** (g mg** **^−1^** ** min** **^−1^** **)**	1.234	1.268	2.385
	**r** **^2^**	0.985	0.988	0.999
Intraparticle diffusion	**k** **_3_** ** (mg g** **^−1^** ** min** **^−1^** **)**	28.016	28.668	24.304
	**C (mg g** **^−1^** **)**	−4.199	14.451	93.679
	**r** **^2^**	0.973	0.968	0.897

**Table 2:** Langmuir and Freundlich isotherm parameters for the adsorption BV14 onto CS/(AM-co-IA)/GO-3.

	CS/(AM-co-IA)/GO-3		
t °C	25	35	45
**Langmuir**			
K_L_ (L mg^−1^)	0.719	0.861	0.980
q_max_ (mg g^−1^)	292.126	339.406	376.429
R_L_	0.218	0.189	0.169
r^2^	0.999	0.999	0.999
**Freundlich**			
K_F_ (L mg^−1^)	185.132	252.101	296.048
n	5.921	7.86	8.663
r^2^	0.887	0.979	0.953

**Table 3:** Thermodynamic parameters for the adsorption BV14 onto CS/(AM-co-IA)/GO-3.

Thermodynamic parameters	ΔH° (kJ kmol^−1^)	ΔS° (J mol^−1^K^−1^)	ΔG° (kJ mol^−1^)		
			298 K	308 K	318 K
CS/(AM-co-IA)/GO-3	12.213	38.318	−81.735	−58.326	−53.406

**Table 4:** Comparison of the adsorption capacity of CS/(AM-co-IA)/GO-3 with different adsorbents reported in the literature for the removal of BV14.

Adsorbent	Maximum adsorption capacity, q_max_ (mg g^−1^)	Kinetic model	Adsorption model	References
poly (AN-co-ST) nanofibers	67.11	Pseudo-second-order	Langmuir-Temkin	[9]
Vegetable residue of Fenugreek-VRF	177.78	Pseudo-first-order	Freundlich	[4]
KMnO4-modified activated carbon	262.39	Pseudo-second-order	Langmuir	[39]
H. verticillata plant biomass	22.22	-	Freundlich	[10]
Activated carbon from Delonix regia pods	141.68	Pseudo-second-order	-	[8]
Calgon carbon (CC)	95.23	Pseudo-second-order	Langmuir	[16]
Curcuma angustifolia scales (CA)	208.33	Pseudo-second-order	Langmuir	[16]
Bottom ash	6.39	Pseudo-second-order	Langmuir- Freundlich	[50]
Deoiled soya	12.03	Pseudo-second-order	Langmuir- Freundlich	[50]
CS/(AM-co-IA)/GO-3 nanocomposite hydrogel	276.21	Pseudo-second-order	Langmuir	This study
